# Sedation in non-invasive ventilation: do we know what to do (and why)?

**DOI:** 10.1186/2049-6958-9-56

**Published:** 2014-11-04

**Authors:** Dan Longrois, Giorgio Conti, Jean Mantz, Andreas Faltlhauser, Riku Aantaa, Peter Tonner

**Affiliations:** Université Paris-Diderot, Hôpitaux Universitaires Paris Nord Val de Seine, Département d’Anesthésie Réanimation Chirurgicale, Hôpital Bichat-Claude Bernard, Paris, France; Department of Intensive Care and Anesthesiology, Università Cattolica del Sacro Cuore, Rome, Italy; Anesthesiology Department, Beaujon Hospital, AP-HP, Université Paris-Diderot, Paris, France; First Department of Internal Medicine, Kliniken Nordoberpfalz AG, Klinikum Weiden, Weiden, Germany; Department of Anesthesiology, Intensive Care, Emergency Care and Pain Medicine, University of Turku, Turku, Finland; Department of Anesthesiology and Intensive Care Medicine, Emergency Medicine Hospital Links der Weser GmbH, Bremen, Germany

**Keywords:** Agitation, Benzodiazepines, Delirium, Dexmedetomidine, Dyspnoea, Ketamine, Non-invasive ventilation, Opioids, Propofol, Sedation

## Abstract

This review examines some of the issues encountered in the use of sedation in patients receiving respiratory support from non-invasive ventilation (NIV). This is an area of critical and intensive care medicine where there are limited (if any) robust data to guide the development of best practice and where local custom appears to exert a strong influence on patterns of care.

We examine aspects of sedation for NIV where the current lack of structure may be contributing to missed opportunities to improve standards of care and examine the existing sedative armamentarium. No single sedative agent is currently available that fulfils the criteria for an ideal agent but we offer some observations on the relative merits of different agents as they relate to considerations such as effects on respiratory drive and timing, and airways patency. The significance of agitation and delirium and the affective aspect(s) of dyspnoea are also considered.

We outline an agenda for placing the use of sedation in NIV on a more systematic footing, including clearly expressed criteria and conditions for terminating NIV and structural and organizational conditions for prospective multicentre trials.

## Introduction

Use of non-invasive ventilation (NIV) in critical and intensive care has expanded greatly in recent decades in response to evidence of its benefits as a means of reducing dependence on invasive (i.e. with tracheal intubation) ventilation and associated complications, and for the management of acute respiratory failure. Adoption and application of NIV are nevertheless still in a phase of evolution [1]. We examine this subject with specific reference to the use and impact of sedation during NIV.

This essay examines a non-exhaustive series of questions about NIV and the possible role of sedation as a co-intervention to NIV. The answers - to the extent that answers are available - emerge from sources that range from evidence-based medicine to expert opinion. This range is itself indicative of the incomplete nature of our current understanding of these matters.

## Review

### Is sedation universal or mandatory during NIV?

A non-exhaustive inspection of recent reports invites the conclusion that we as physicians are either indifferent to or unconcerned about the general topic of sedation during NIV. The authors of the most recent Cochrane review on the place of NIV as a weaning strategy for intubated adults with respiratory failure reported that only one of the studies they identified used a standardized sedation protocol before or after initiation of NIV (Table 1) and argued that the role of sedation as a co-intervention requires specific investigation in future trials [2–8]. Separately, Scala has identified only eight small clinical studies of sedation in NIV, in which a total of 183 patients were assigned to seven different sedative drug regimens, the effects of which were assessed by multiple different and non-congruent outcome parameters (Tables 2 and 3) [9–17].

**Table 1:** **Summary of studies making any report of sedation use in the context of NIV in adults with critical illness (compiled from Burns et al.** [2] **)**

Study	No. of patients	Patient characteristics	Experimental NIV strategy	Sedation status
Hill et al. 2000 [4]	21	ARF	VPAP in ST-A mode	Sedation protocol reportedly used. Study published as abstract only
Nava et al. 1998 [5]	50	Exacerbation of COPD; mechanical ventilation for at least 36–48 h	Non-invasive pressure support on conventional ventilator delivered with face mask	Sedation reportedly used during NIV but apparently not protocolized or defined
Prasad et al. 2009 [6]	30	COPD; AHRF	Bilevel NIV (pressure mode) delivered by full face mask	Patients received neuromuscular blocking drugs and sedatives in immediately preceding phase of invasive ventilation. Use of sedation during NIV not clear
Rabie Agmy et al. 2004 [7]	37	Exacerbation of COPD	Proportional-assist NIV in timed mode, delivered by face or nasal mask	Patients received neuromuscular blocking drugs and sedatives in immediately preceding phase of invasive ventilation. Use of sedation during NIV not clear
Vaschetto et al. 2012 [8]	20	Hypoxaemic respiratory failure; invasive mechanical ventilation for at least 48 h	Helmet NIV	Sedation reportedly used during NIV but apparently not protocolized or defined. Rates of continuous sedation during NIV reported to be similar in both groups (*a priori* study outcome)

In all the studies, the control strategy used was invasive pressure support.

ARF, acute respiratory failure; AHRF, acute hypercapnic respiratory failure; ST, spontaneous/timed; VPAP, variable positive airway pressure.

**Table 2:** **Studies of sedation in NIV (compiled from Scala** [9] **)**

Study	No. of patients	Indication	NIV interface	Type of sedative	Initiation of sedation
Rocker et al. [10]	10	ARF	FFM	Morphine	At start of NIV
Constantin et al. [11]	13	ARF (n = 10); AHRF (n = 3)	FFM	Remifentanil, midazolam*	Poor NIV acceptance
Rocco et al. [12]	36	ARF	FFM, helmet	Remifentanil	Poor NIV acceptance
Akada et al. [13]	10	ARF	FFM	Dexmedetomidine^†^	Poor NIV acceptance
Takasaki et al. [14]	2	SAA	FFM	Dexmedetomidine	Poor NIV acceptance
Clouzeau et al. [15]	10	ARF (n = 7); AHRF (n = 3)	FFM	Propofol	Poor NIV acceptance
Senoglu et al. [16]	40	COPD	FFM	Dexmedetomidine (n = 20); midazolam (n = 20)	At start of NIV
Huang et al. [17]	62	ACPO	FFM, helmet	Dexmedetomidine (n = 33); midazolam (n = 29)	Poor NIV acceptance

The last two rows identify randomized controlled trials; other trials were reported not to have been controlled. See also Table 3 of this review.

ACPO, acute cardiogenic pulmonary oedema; ARF, acute respiratory failure; AHRF, acute hypercapnic respiratory failure; FFM, full face mask; SAA, severe asthma attack.

*Combined with propofol in three cases. ^†^Combined with morphine in one case and with propofol in one case.

**Table 3:** **Reported dosages of sedatives administered in studies of sedation in NIV (compiled from Scala** [9] **)**

Drug	No. of patients	Dosage	Sedation target range
Dexmedetomidine	41	1 μg/kg (bolus); 0.2-0.7 μg/kg/h (infusion)	RSS 2-3; RASS 2-4; BIS >85
Midazolam	41	0.05 mg/kg (bolus); 0.05-0.1 mg/kg/h (infusion)	RSS 2-3; RASS 2-4; BIS >85
Remifentanil	38	0.025-0.1 μg/kg/min (infusion)	RSS 2-3
Propofol	43	0.4 μg/mL (target serum concentration; step-down to 0.2 μg/mL)	OAAS/S 3-4

The information in this Table is derived from references 10-17. See also Table 2 of this review.

BIS, bispectral index; OAAS/S, Observer’s Assessment of Alertness/Sedation Scale, RASS, Richmond Agitation and Sedation Scale; RSS, Ramsey Sedation Scale.

The drift of these observations appears to be that either sedation has not been perceived as a major problem or opportunity within the wider context of NIV use or that it has not been studied systematically. The first of these positions may have led to the second; alternatively, the lack of studies may reflect complaisance (if not complacency) about this aspect of NIV technique and procedure. In any event, both these positions suggest a lack of attention to the possibilities in this area of NIV.

Narrowly considered, therefore, the answer to our own question must be “We do not know”. However, the findings of the only survey of practice that we have identified make it clear that in reality the answer is “No”, as only ≈ 25% of NIV patients received sedation (rising to ≈ 40% in critical care) [18]. Whether or not that percentage represents an appropriate level of sedation use among recipients of NIV is just one of many questions to which there is at present no definitive answer. Inspection of Table 2 suggests that, in those studies where sedation during NIV was explicitly addressed, the main reason for sedation was “poor NIV acceptance”. One general aspect of poor NIV acceptance could be related to the patient-device interface, i.e. the type of mask that is used and also the assisted ventilation pattern. The choice of NIV interface may influence the need for sedation. While it can be argued that “the clinical efficacy of different masks is on average very similar” [19], our experience is aligned with reports that patient acceptance is greatest with the least constricting interfaces -such as the helmet - and declines as more intrusive forms of mask are used [20]. As for the breathing modes, bilevel positive airway pressure often produces a need for anxiolysis or sedation whereas spontaneous breathing patterns such as continuous positive airways pressure seldom require such interventions.

In summary, sedation is not mandatory for NIV but it may help in specific situations. There are at present no explicit guiding principles or simple formulae to identify those situations.

### Does NIV affect sedation goals?

The goals of sedation for a cooperative patient in the intensive care unit (ICU) are to provide analgesia and comfort, preserve day/night cycles (including natural sleep) and avoid nuisances such as ambient light and noise. Additional goals include haemodynamic stability, preservation of metabolic homeostasis, muscular relaxation, preservation of diaphragmatic function and attenuation of the stress/immune response, as well as considerations such as programmed withdrawal from sedation: they should be no different during NIV.

Nevertheless, if we regard NIV as a stage in the progression from intermittent mandatory ventilation to spontaneous breathing then there must be a parallel expectation of a progressive reduction in use of sedation. Two other points need to be discussed for sedation during NIV. Firstly, the avoidance of the respiratory depressant effects of different sedatives; secondly, untoward effects of sedative drugs on the upper airway, a topic that has received new impetus with increased awareness of obstructive sleep apnoea [21–24].

Therefore the answer to this question is “Yes”, both quantitatively (i.e. less ‘deep’ sedation) and qualitatively in that sedation during NIV must be performed with no/minimal respiratory depression and no/minimal impairment of the upper airway.

### Is sedation *per se*a factor contributing to the success or failure of NIV?

Patient acceptance and compliance are essential to the success of NIV [13]. Achieving patient acceptance and compliance is a multifaceted exercise, with staff proficiency and competence being one major influence [25]. However, acceptance/compliance also relate directly to sedation since neither can be expected from an insensate patient; nor are they likely to be forthcoming if the patient is anxious, agitated or disoriented. The necessity of a sedation regimen that brings the patient to a state of calm, alert cooperation is clearly implied by these considerations.

**Figure 1:**
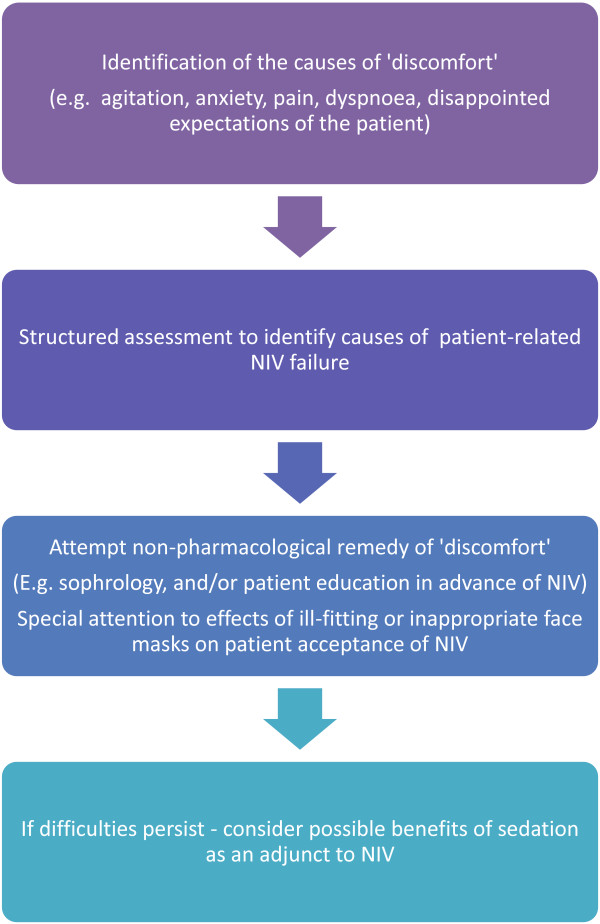
**Clinical reasoning pathway for the use of sedation in NIV.**

Any decision to resort to sedation must be taken as the last stage in a careful evaluation of the causes of actual or pending failure, as outlined in Figure 1. It must also take account of the fact that the likely success of NIV in hypoxaemic respiratory failure varies considerably according to the presenting condition [26] and that there is no robust evidence that sedation will materially affect situations where the response rate to NIV is intrinsically poor. Indeed, adding sedation in these situations may be disadvantageous by obscuring a failure of NIV due to underlying pathology and thus delaying a necessary intubation. Similarly, sedation does not obviate any of the contraindications to NIV [26].

Nava and Ceriana [25] have partitioned NIV failure into immediate (< 1 h after commencement), early (1–48 h) and late (> 48 h) and identified predictors of failure for each time segment. Factors implicated in immediate NIV failure include “intolerance, agitation, and patient–ventilator asynchrony”, for which “judicious sedation” is recommended but not characterized in detail.

Sleep disruption was identified as a factor in late NIV failure [25]. The subject of sleep and sleep disruption in ICU patients has attracted much research in recent years (see for example Cabello et al. [27] and Roche Campo et al. [28]) and one instinctively inclines to the belief that more normal patterns and quality of sleep are part of the restoration of health. Whether this is indeed the case is harder to demonstrate than might be expected but investigations of the differential effects of sedatives on electrophysiological dimensions of sleep provide food for thought [29]. This is, of course, an area where environmental alterations (i.e. minimizing noise and disturbance) may also have an important influence and such measures should always precede any use of sedatives.

In mechanically ventilated patients, ventilator asynchrony may adversely affect sleep [30]: the extent to which this disruptive interaction may work in the opposite direction is not clear, but there is evidence that, in NIV patients, sleep is associated with more asynchrony and blood-oxygen desaturations than the awake state [31]. These data were obtained from a small cohort of patients receiving long-term NIV at home and probably do not fully illuminate the situation of ICU patients receiving both NIV and sedatives. Choice/type of ventilator is also an important influence on asynchrony [32, 33] and, at least in some cases, is likely to be more significant than use or non-use of sedatives. Similarly, ventilators with specific leak compensation modes and the ability to generate flows ≥ 10 L/min provide better patient compliance than ICU ventilators with little leak adaptive capacity.

The answer to the question is thus that we do not know for certain but that it is unlikely that sedation can ‘rescue’ poorly configured or inappropriate NIV. However, we hypothesize that adequate sedation can improve patient comfort in specific situations (see below).

### What types of patients/specific situations might benefit from sedation during NIV?

Reference to English language guidelines for NIV [1] reveals that the range of indications for which there is compelling or even strongly persuasive (Grade 2B or better) evidence for benefit of NIV is small and may be summarized as follows.

*Acute respiratory failure in the forms of*:

- Acute-on-chronic exacerbation of chronic obstructive pulmonary disease (COPD) with acidotic and hypercapnoeic features
- Respiratory failure secondary to cardiogenic pulmonary oedema not arising from shock or acute coronary syndrome
- Acute respiratory failure in immunocompromised patients.

*As an adjunct to extubation (but only in expert centres) for*:

- Patients who have COPD
- Patients considered to be at high risk of recurrent respiratory failure.

We would expect most candidates for sedation during NIV to come from these categories, and hence to have certain common presenting features.

As noted above, discomfort, anxiety, agitation, pain, dyspnoea and the disappointed expectations of the patient are central in many cases to failure of NIV and hence also to the decision to use sedation in NIV. Delirium may also be a consideration. Use of sedation in NIV is, in this context, firmly within the sphere of the clinical practice guidelines for the management of pain, agitation, and delirium [34] and many of the provisions of those guidelines are relevant, most especially the obligation to identify and correct (by non-pharmacological means if feasible) the causes of pain, agitation, etc.

### Agitation and delirium

A first category to consider is patients who are already agitated before tracheal extubation. A systematic investigation of the causes of anxiety should precede any prescription of a sedative drug. If all otherwise correctable causes of anxiety are eliminated, and anxiety *sine materia* or anxiety due to a decrease/change in sedative regimen is diagnosed, a case may be made (albeit on the basis of very slender clinical experience) for dexmedetomidine as an aid to extubation [35]. The mechanisms underlying this benefit are not clear and require further, systematic investigation.

The impact of delirium in patients receiving NIV is urgently in need of attention. The most substantial report we have found identified a high prevalence of delirium in NIV patients (≈37%) and linked that to a marked increase in risk of NIV failure. However, the data on which these findings were based were described as “scarce and of low quality” [36]. The role of sedation in the promotion or prevention of ICU delirium has attracted much comment in recent years but firm conclusions are still hard to come by. For the moment, we are unable to go beyond the pain, agitation, and delirium guidelines, which offer weak (Grade 2B) endorsement for dexmedetomidine in delirium management. “Prevention*”* of delirium rests substantially on non-pharmacological methods, particularly early mobilization. Where sedation is used at all, an emphasis on early mobilization implies a sedative regimen that facilitates patient participation. (See Nydahl et al. [37] for a recent perspective on this matter).

### Dyspnoea

A second category of patients could be those who are dyspnoeic and anxious, dyspnoea being associated with delay of extubation [38]. The neuro(patho)physiology and clinical aspects of dyspnoea have been examined in detail in a report of the American Thoracic Society that stratifies dyspnoea according to the quality of the dyspnoea experience, the stimuli that evokes it and the afferent neuronal pathways that mediates it [39].

A full discussion of this matter is beyond the scope of this review but it is worth noting that there is an affective component of dyspnoea that may be differentiated from the sensory dimension and might be amenable to independent manipulation [40–43]. This signals the importance of identifying and appraising the anxiety component of dyspnoea. Self-evidently, the cooperation of the patient is needed for this and any existing sedation regimen must be adapted to that need.

The affective dimension of dyspnoea may be investigated using either single-item ratings of severity of distress or unpleasantness or multi-item scales of emotional responses such as anxiety [39, 44, 45]. However, there are a vast array of dyspnoea rating scales, which address different aspects of the condition. This is not a reason not to measure dyspnoea but it is essential to specify which scale is used for the purpose and to recognize that the nature of any intervention on dyspnoea is likely to be determined (or at least influenced) by the aspects of dyspnoea privileged by the chosen scale.

Hence, sedation could be of benefit in situations where NIV is clearly indicated and where careful evaluation identifies anxiety, dyspnoea with a high affective dimension or delirium as barriers to its successful implementation.

### Are there evidence-based reasons to prefer specific sedative drugs during NIV?

There are no robust data to favour any one drug, class of drugs or protocol over all others. Some of the criteria that may shape the selection of sedatives for NIV are summarized in Table 4. No drug or class fully satisfies all these criteria and the final decision rests for the moment on a sequence of clinical reasoning.

**Table 4:** **Properties of sedative drug classes relevant to delivery of sedation in NIV**

Sedative	Haemodynamic stability	Analgesia	Amnesia	Anxiolysis	PVD	Avoidance of PONV	Promotion of natural sleep	Suitability for use after extubation	Delirium avoidance	Total
**Propofol**	2	2	2	2	2	4	2	2	1	20
**Midazolam**	3	2	4	2	2	2	2	1	1	19
**Opioids**	4	4	1	2	1	1	1	2	1	20
**Dexmedetomidine**	3	2	2	4	4	2	4	4	3	28
**Ketamine**	4	3	2	1	4	1	1	4	1	21

Larger numbers indicate a more satisfactory impact on the nominated property. This is primarily a qualitative and relative assessment of the features and benefits of different drugs and drug classes, framed in general terms. Hence, the individual category scores and in particular scores shown in the ‘Total’ column are crude summaries that should not be over-interpreted and which do not necessarily reflect the net merits or demerits of particular agents in the circumstances of a particular patient.

PONV, postoperative nausea and vomiting; PVD, preservation of ventilatory drive.

We fully endorse the general presumption against benzodiazepines expressed in the pain, agitation, and delirium guidelines. It is, therefore, a matter of concern that the data of Devlin et al. suggest that benzodiazepines retain a hold in NIV [18]: however, those data are 7 years old and may not represent current habits. Fresh research into prescribing patterns (and the reasoning underpinning them) is desirable: study of the effects of switching sedatives would also be illuminating. Patterns of sedation when NIV is delivered on standard wards are undocumented as far as we know and this is another area that deserves more attention.

Given the pathophysiology of NIV failure, at least three aspects could be influenced by the choice of sedative drugs: the patency of the upper airway, respiratory depression and the affective dimension of dyspnoea. From a pharmacological point of view, dexmedetomidine appears to offer the range of qualities best configured to address these concerns (see for example Hsu et al. [46]). However, the evidence from controlled trials is not sufficient to give this conclusion the force of a guideline and additional considerations (see Table 4) may shape the final selection of drugs for individual patients. (See also Ho et al. [47] for a cautionary case report).

Effects on airways patency and the timing and drive of respiration have been examined earlier in this review and make a case against benzodiazepines (and perhaps other γ-aminobutyric acid-ergic agents) and possibly in favour of dexmedetomidine. Opioids and benzodiazepines decrease upper airway diameter and are probably deleterious during NIV. Propofol has also been shown to increase the collapsibility of the upper airway in a dose/concentration-dependent manner [23].

Dexmedetomidine has no direct effects on the patency of the upper airways. When used as adjunct therapy it may reduce requirements for opioids (or other sedatives) and so reduce the likelihood of opioid-induced compromise of the upper airways. Among the sedative drugs, dexmedetomidine has the lowest risk of depression of the respiratory centres.

Ketamine does not cause respiratory depression at doses given for analgesia or procedural sedation [48]. Furthermore, it decreases airway resistance, improves dynamic compliance and preserves functional residual capacity, minute ventilation and tidal volume, while retaining protective pharyngeal and laryngeal reflexes [49]. Ketamine can produce hypersalivation and emergence reactions [49]. Because of its effects on the sympathetic nervous system, ketamine should not be used in decompensated heart failure (typically cardiogenic pulmonary oedema in the context of NIV). There is a relatively abundant literature concerning the use of ketamine for procedural sedation [50], but experience for sedation during NIV is practically non-existent.

The answer to this question, therefore, is that sedative drugs used during NIV should have properties that further the goals of sedation during NIV.

From a pharmacological point of view, benzodiazepines should be avoided: outcome studies in mechanically ventilated patients are consistent with that view but there are no similar trials in NIV to support definitive guidance [51]. (Other recent work provides a timely reminder that without a robust experimental structure it is difficult to make meaningful comparisons between sedatives [52]).

Dexmedetomidine and ketamine seem to have the most suitable overall pharmacological profiles. Propofol and opioids (such as remifentanil) are in an intermediate position.

## Conclusions

Much of the use of sedation in NIV appears to be empirical and is perhaps unstructured. Above all, it appears to be under-researched: we may be doing better than we realize or we may be doing less well than we could. With the continuing growth of NIV as a clinical resource there is a need for sedation practice to be put on a more systematic footing.

To that end we propose a three-point plan.

1. Repeat the international survey of 2007 [18] to ascertain how, if at all, patterns of sedation use in NIV have changed in the intervening years and, if possible, identify the drivers of change.
2. Review best practice frameworks in NIV to ensure that guidance includes indications for sedation use, standardized sedation protocols and clearly expressed criteria and conditions for terminating NIV.
3. Develop at least one (preferably more) prospective multicentre trial on the effects of sedation in NIV. Such a trial needs to enrol substantially more patients than have hitherto been recruited to such studies and needs to incorporate the principles identified in point (2) and a set of unambiguous, informative and reliable endpoints, including a predefined and generally accepted definition of NIV failure. The work of Huang et al. [17] provides a useful, though not exhaustive, template in this respect.

In addition to standardization of sedation protocols, such a trial needs to apply consistent and predefined NIV modalities. Given the need for substantial numbers of patients, we anticipate that such a study would, in the first instance, be confined to patients with acute exacerbation of COPD, as this is the only category likely to yield sufficient patients within an acceptable and practicable period of time.

An alternative or additional possibility would be a prospective trial of NIV to prevent acute respiratory failure in patients recovering from cardiac, abdominal or possibly thoracic surgery [53, 54]. Given that these patients would be emerging from surgical anaesthesia, there might be a larger role for sedation and that would facilitate rapid recruitment.
